# Supplementary material for: Defective mitochondrial COX1 translation due to loss of COX14 function triggers ROS-induced inflammation in mouse liver
Source: Nat Commun. 2024 Aug 12;15:6914. doi: 10.1038/s41467-024-51109-y (PMC11319346; doi:10.1038/s41467-024-51109-y)
Supplement: Supplementary file 1 — Supplementary Information [file 41467_2024_51109_MOESM1_ESM.pdf]

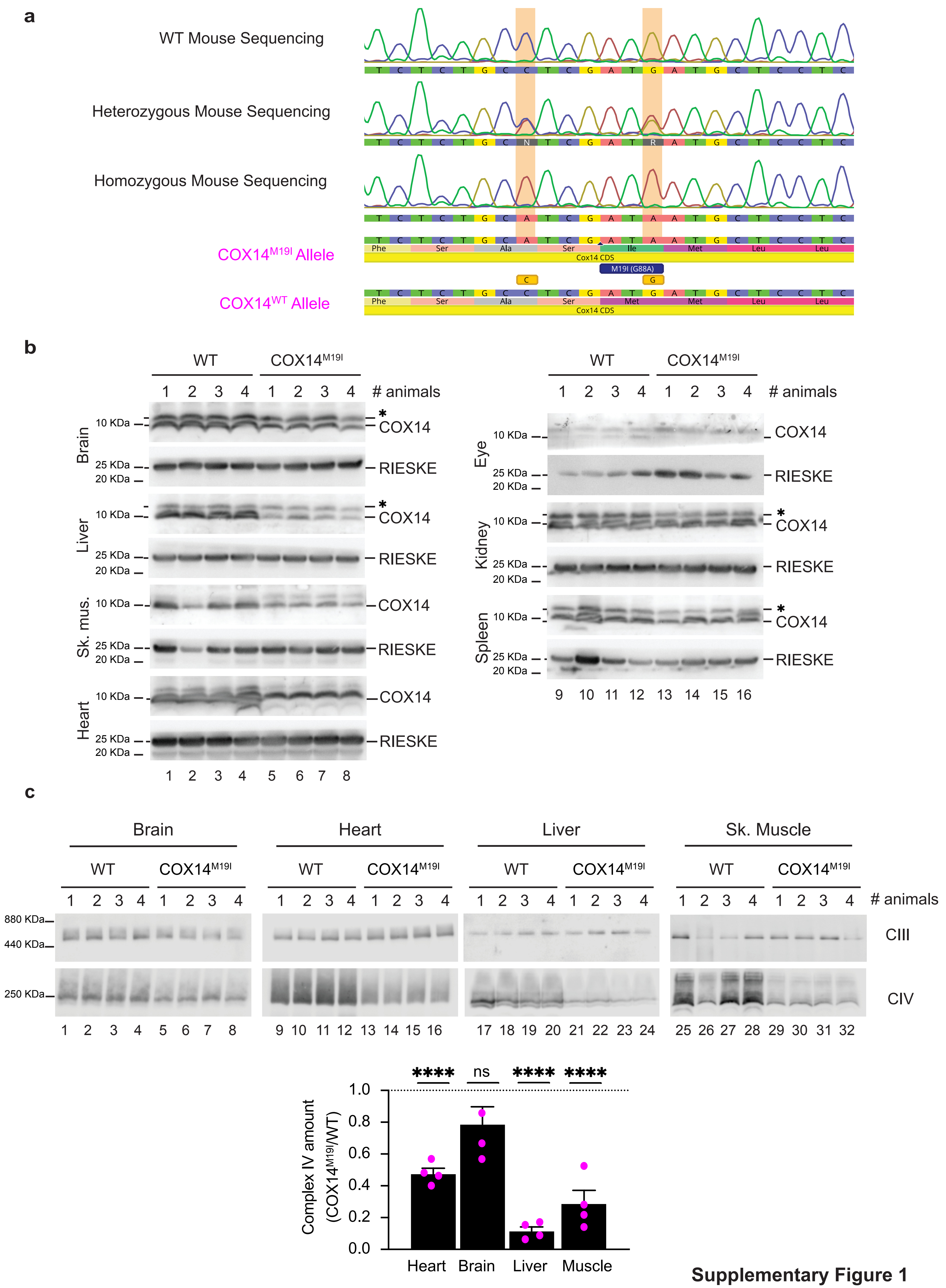

Supplementary Figure 1

Supplementary Figure 1. Altered protein pattern in COX14^{M19I} mice

a Multiple sequence alignment of sequencing reads of the *Cox14* allele from wild-type (WT), heterozygous and homozygous COX14^{M19I} mice. **b** Western blot analysis of tissue lysates from WT and COX14^{M19I} mice tissues with indicated antibodies. See quantification in Figure 1c, n=4. **c** Isolated mitochondria from indicated tissues of 24 week old mice, solubilized in 1% N-Dodecyl b-D-maltoside (DDM) and analyzed by BN-PAGE and western blotting to detect respiratory chain complex III (CIII) and complex IV (CIV) using antibodies against RIESKE and COX1 respectively, Means ± SEM, n =4; Unpaired t test; ns = non-significant, $p < 0.0001$. Quantification presented at the bottom. Source data are provided as a Source Data file.

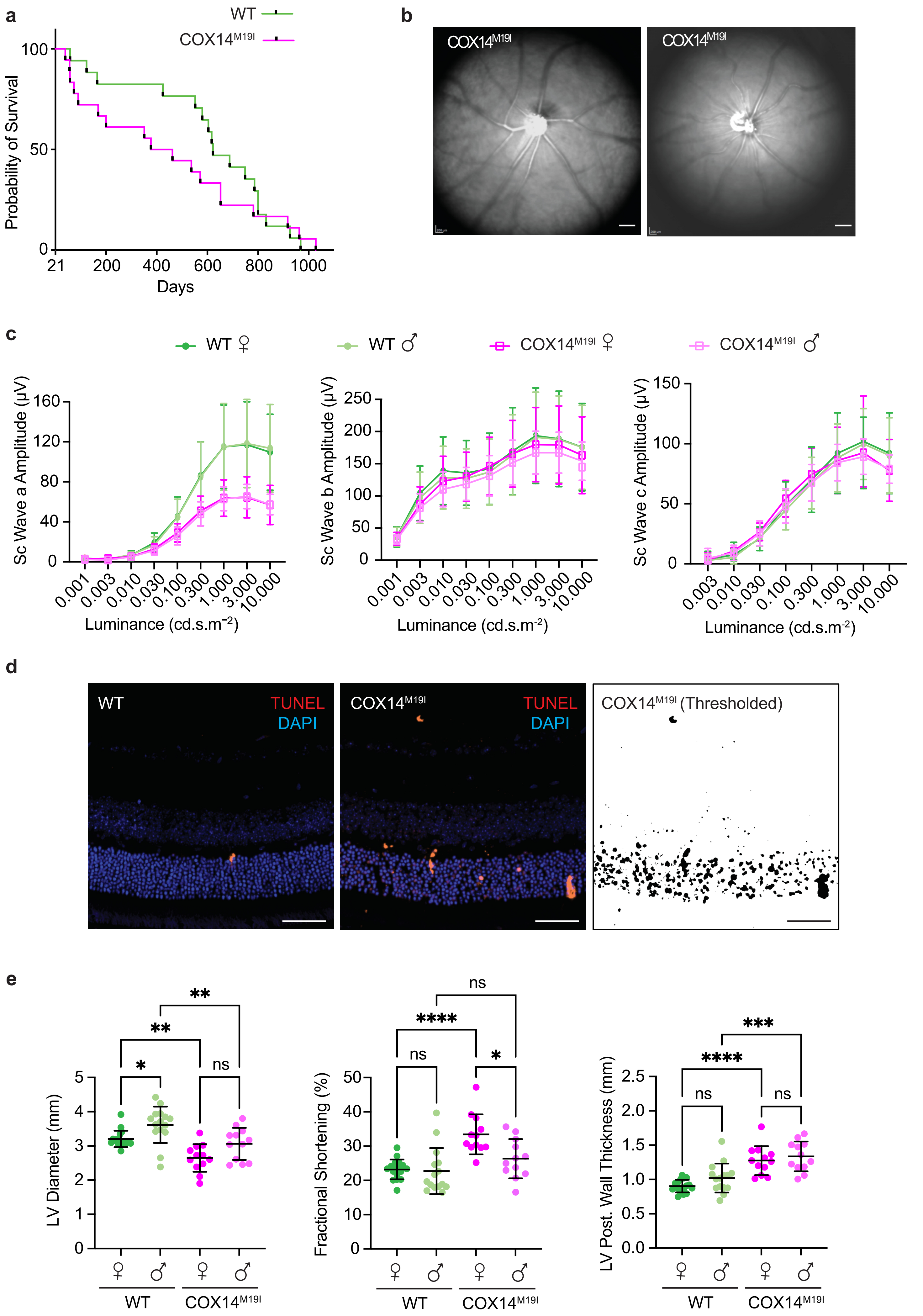

Supplementary Figure 2

Supplementary Figure 2. Pathophysiology of COX14^{M19I} mice

a Survival curve of WT and COX14^{M19I} male mice. **b** Representative fundus images from COX14^{M19I} eyes. Scale bar 400μm. **c** ERG Amplitude measurements as a response to increase in luminance in WT and COX14^{M19I} males (M) and females (F) showing the scotopic waves, a (rod cells), b (ON-bipolar cells) and c (pigmented epithelium cells recorded in 30 week old animals**. d** Retina of WT and COX14^{M19I} mice showing TUNEL positive cells (orange) in the outer nuclear layer (ONL) and the inner nuclear layer (INL) at 30 weeks of age, n = 3. Scale bar 50μm. **e** Echocardiography (left panel, left ventricle diameter; middle panel, fractional shortening, right panel, left ventricle posterior wall thickness) of WT and COX14^{M19I} mice. Means ± SD, n=12, One-way ANOVA, ns=non-significant, left panel: WT females vs. WT males $p = 0.0361$; WT females vs. COX14 ^{M19I} females $p = 0.0046$; WT males vs. COX14 ^{M19I} males $p = 0.0061$, middle panel: WT females vs. COX14 ^{M19I} females $p < 0.0001$; COX14 ^{M19I} females vs. COX14 ^{M19I} males $p = 0.0111$, right panel: WT females vs. COX14 ^{M19I} females $p < 0.0001$; WT males vs. COX14 ^{M19I} males $p = 0.0003$. Source data are provided as a Source Data file.

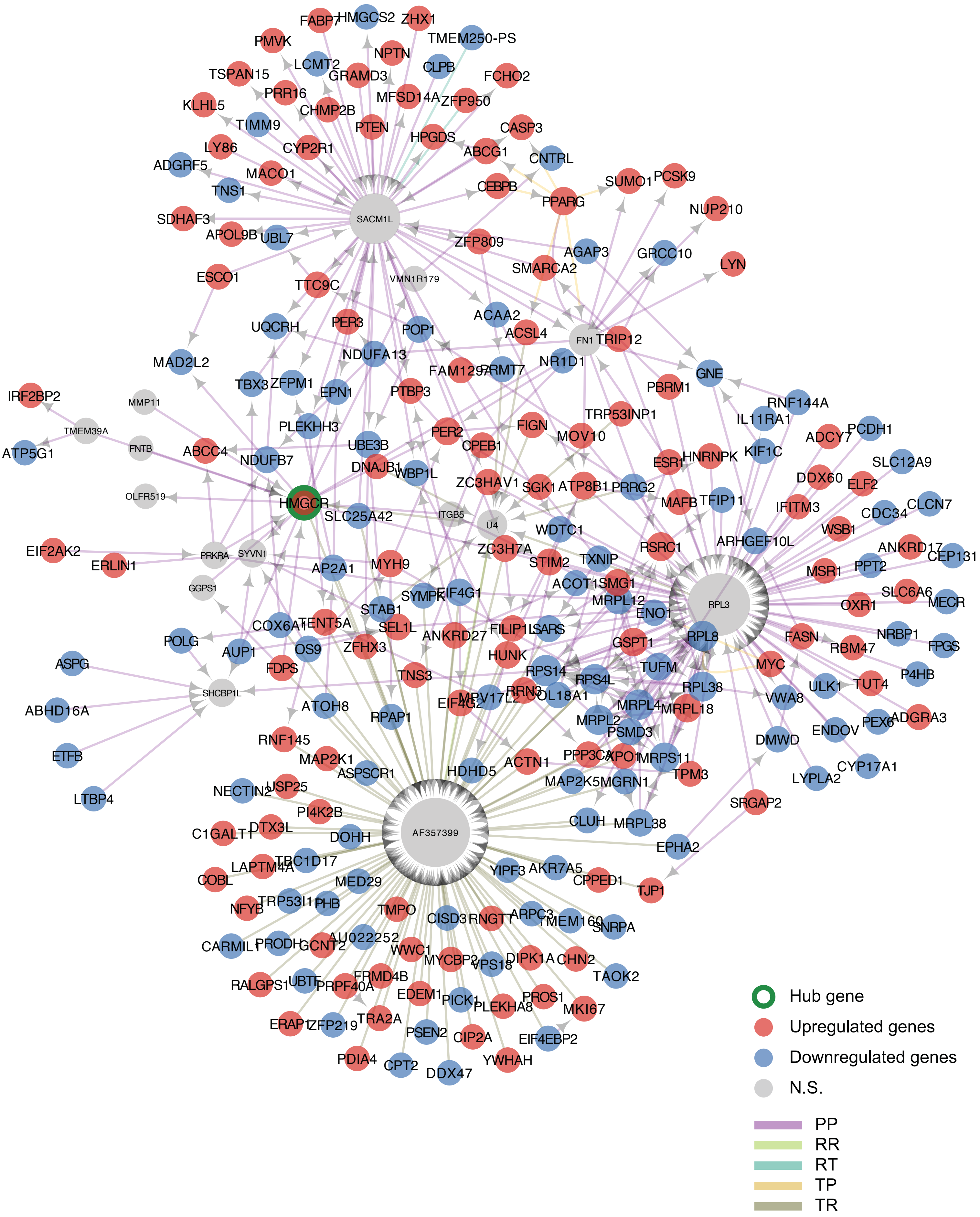

Supplementary Figure 3

Supplementary Figure 3. Interaction network analysis.

Hmgcr interaction network, with first degree (direct) and second degree (neighbours of direct) connections of Hmgcr. (Green node: hub gene (Hmgcr); red nodes: upregulated genes in COX14^{M19I} samples; blue nodes: downregulated genes in COX14^{M19I} samples; grey nodes: interactors in the network which are not significantly up- or downregulated.) The different kinds of (directed) interactions considered were: protein-protein (PP), RNA-RNA (RR), transcription factor-protein (TP), transcription factor-RNA (TR) and RNA-transcription factor (RT) interactions. The size of the nodes denotes the degree (number of outgoing + incoming interactions).

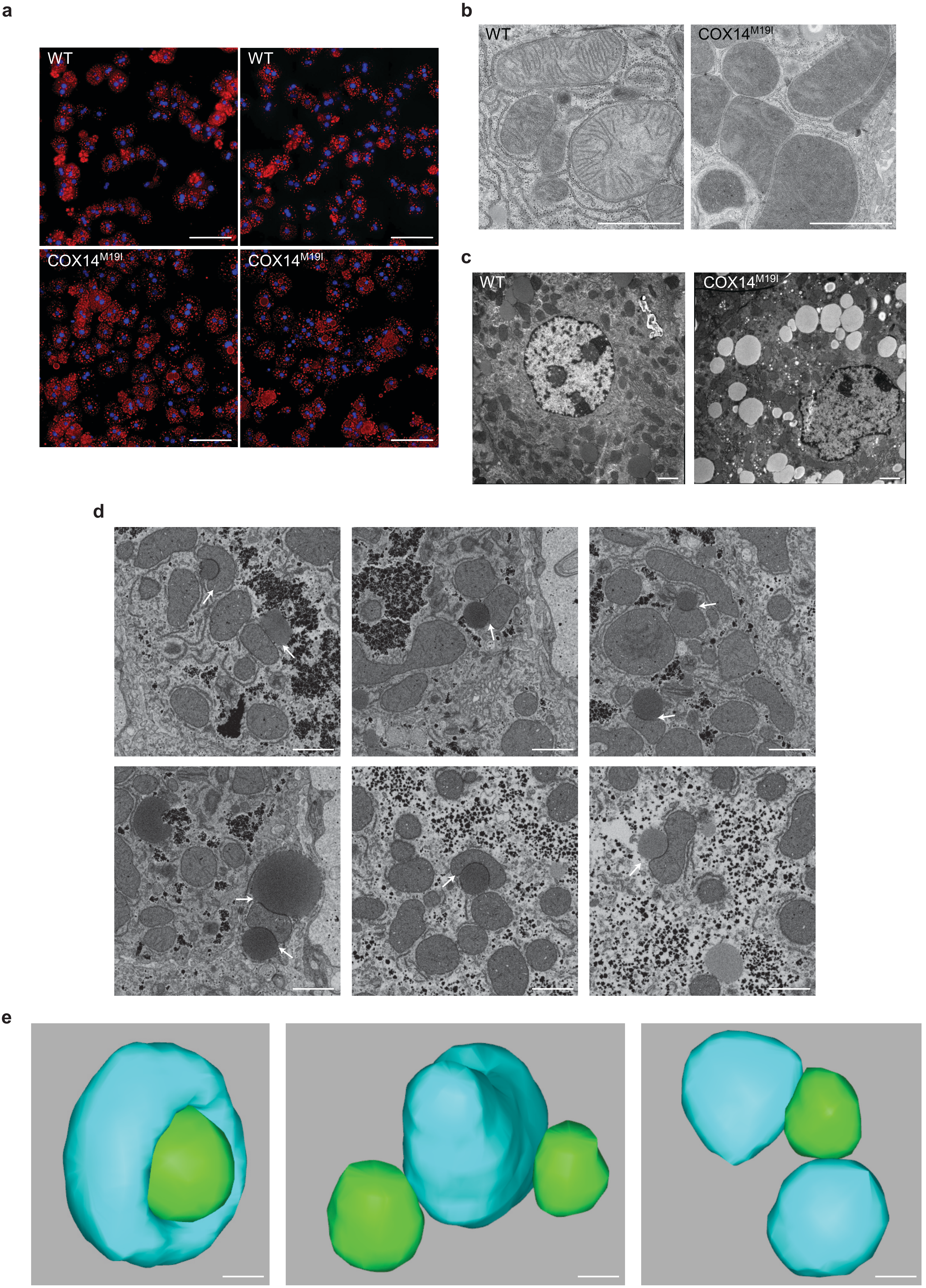

Supplementary Figure 4

Supplementary Figure 4. Altered mitochondrial organization in COX14^{M19I} liver

a Cytochemistry of isolated primary hepatocytes from WT and COX14^{M19I} mice using Oil Red O. Scale bar 100μm. **b** Representative TEM images of isolated primary hepatocytes from WT and COX14^{M19I} mice. Scale bar 500nm. **c** Representative TEM images of liver tissue samples from WT and COX14^{M19I} mice. Scale bar 2μm. **d** Representative FIB-SEM images of liver tissue samples from COX14^{M19I} mice. Scale bar 1μm. **e** Representative 3D reconstructions of interaction between mitochondrion and lipid bodies observed in FIB-SEM images of liver tissue samples from COX14^{M19I} mice. Scale bar 250nm.

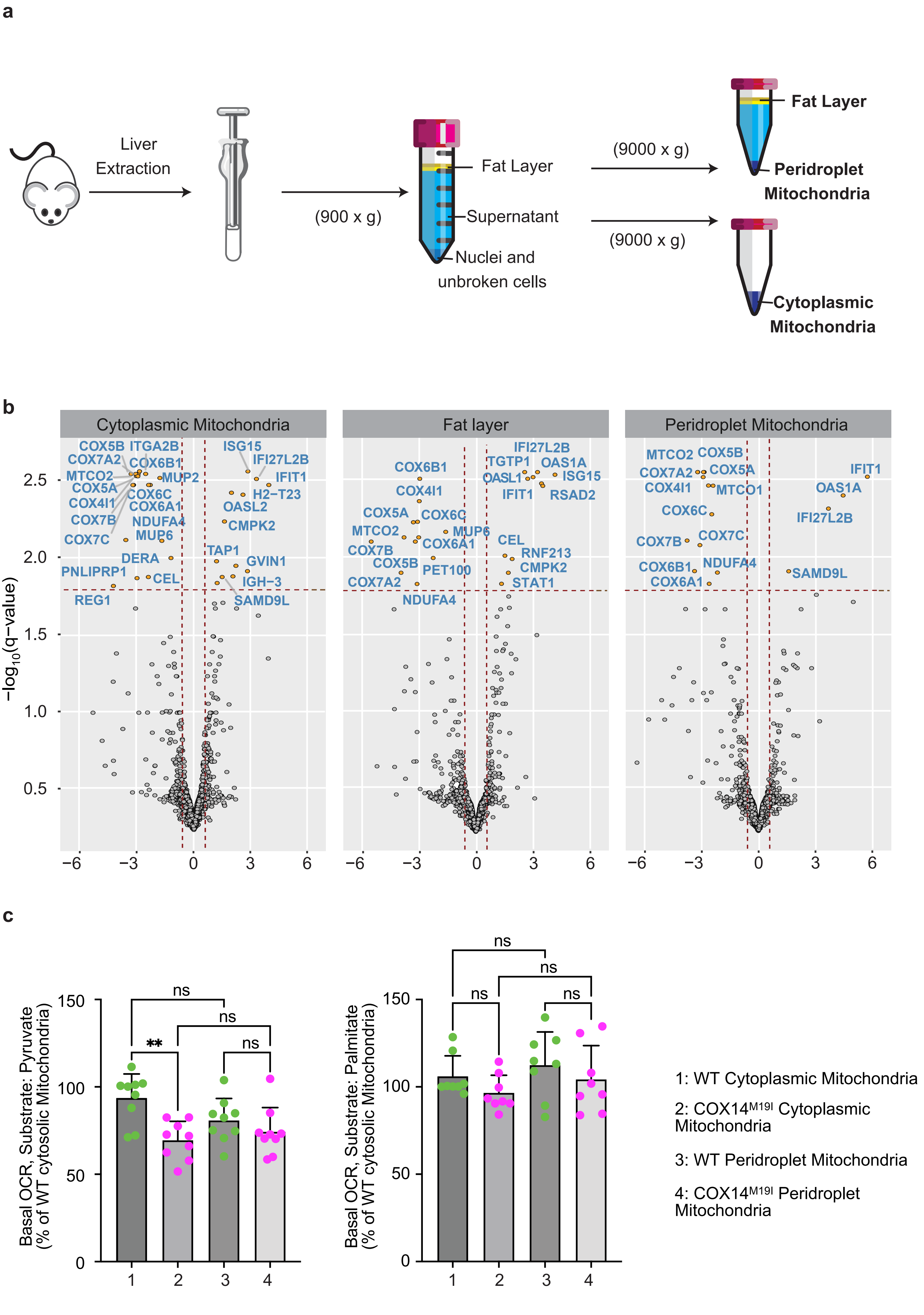

Supplementary Figure 5

Supplementary Figure 5. Analysis of peridroplet mitochondria

a Cartoon depicting the fractionation of mice liver for separation and isolation of different intracellular fractions. The fat layer was washed and subjected to high-speed centrifugation to separate the lipid body associated mitochondria, termed as peridroplet mitochondria, from the fat layer. **b** Volcano plots for mass spectrometric analysis of proteomes from the different intracellular fractions, obtained from WT and COX14^{M19I} mice livers, n=3. **c** Real-time respirometry, using succinate and palmitate as substrates, of cytoplasmic and peridroplet mitochondria obtained from WT and COX14^{M19I} mice livers; oxygen consumption rates, OCR. Means ± SEMs; n=9; One-way ANOVA; ns=non-significant, Pyruvate respiration p = 0.0019. Source data are provided as a Source Data file.

a

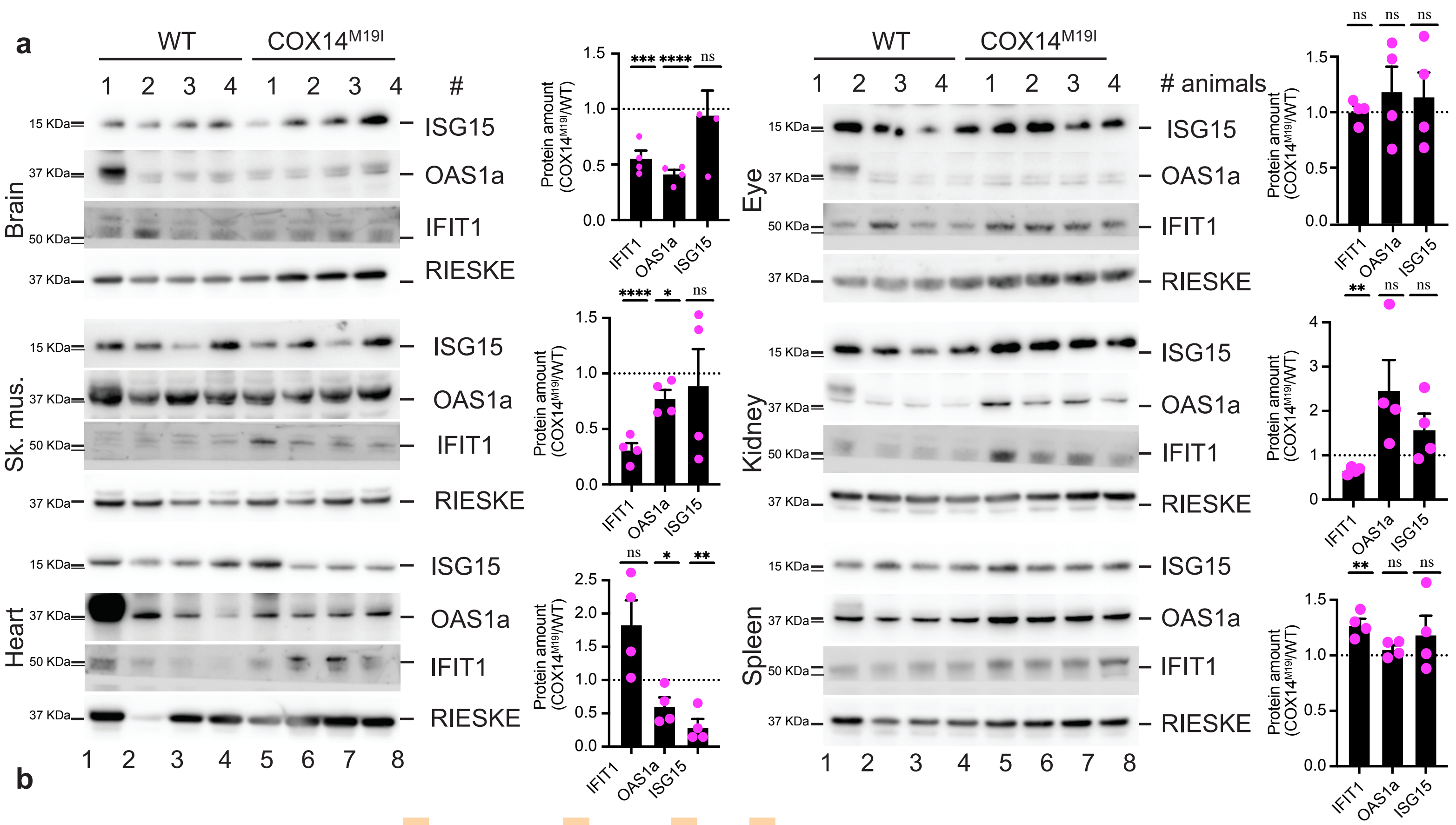

b

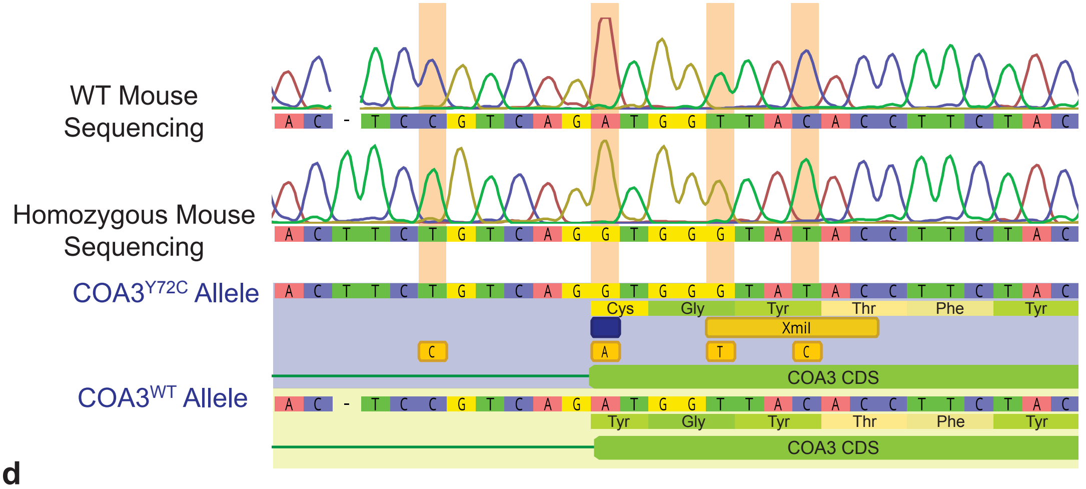

c

Parameters	Change rel. to wt	p value
Albumin	–	0.908
Creatine Kinase	–	0.146
Cholesterol	↑	0.001
AST	–	0.992
ALT	–	0.142
Glucose	–	0.487
Total protein	–	0.947
HDL-Cholesterol	↑	0.001
Creatinine	–	0.632
Sodium	–	0.067

d

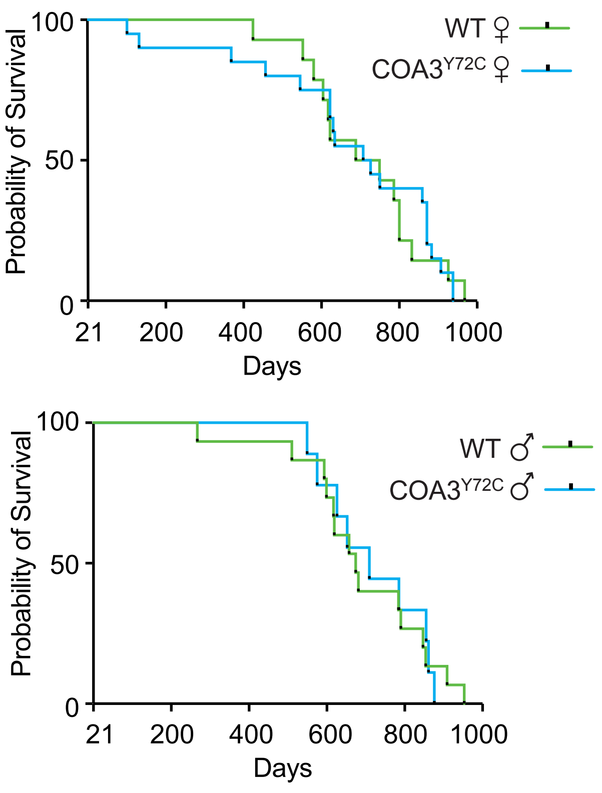

e

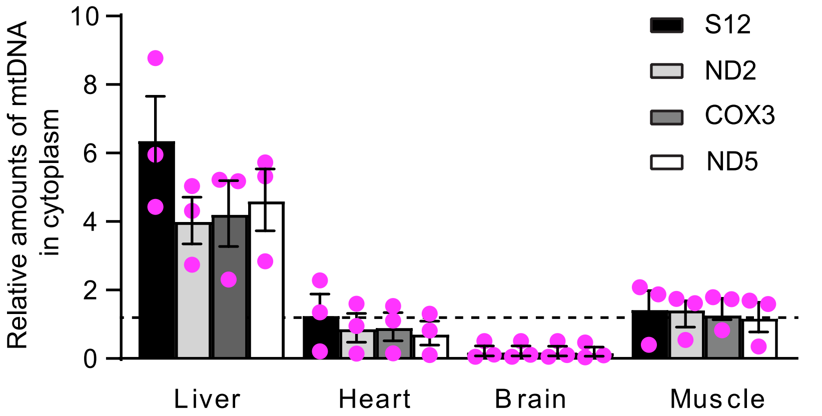

Supplementary Figure 6

Supplementary Figure 6. Inflammatory pathway activation in COX14^{M19I} mouse tissues and characterization of COA3^{Y72C} mice

a Western blot analysis and quantification of indicated tissue lysates from 24-week-old WT and COX14^{M19I} mice using indicated antibodies. Means ± SEMs; n=4; Unpaired t test; ns=non-significant, Brain: IFIT1 p = 0.0009; OAS1a p > 0.0001; Muscle: IFIT1 p > 0.0001; OAS1a p = 0.0246; Heart: OAS1a p = 0.0258; ISG15 p = 0.0011; Kidney: IFIT1 p = 0.0002; Spleen: IFIT1 p = 0.0028. **b** Multiple sequence alignment of sanger sequencing reads for the *Coa3* allele from wild-type (WT) and homozygous COA3^{Y72C} mice. **c** Serum biochemical parameters for COA3^{Y72C} mice. Arrows indicate increase compared to WT with p values. **d** Survival curve for WT and COA3^{Y72C} female and male mice. **e** Measurement of relative DNA abundance in the cytosolic fractions from WT and COX14^{M19I} mice livers fractionation samples. Means ± SEM, n = 4. Source data are provided as a Source Data file.

a

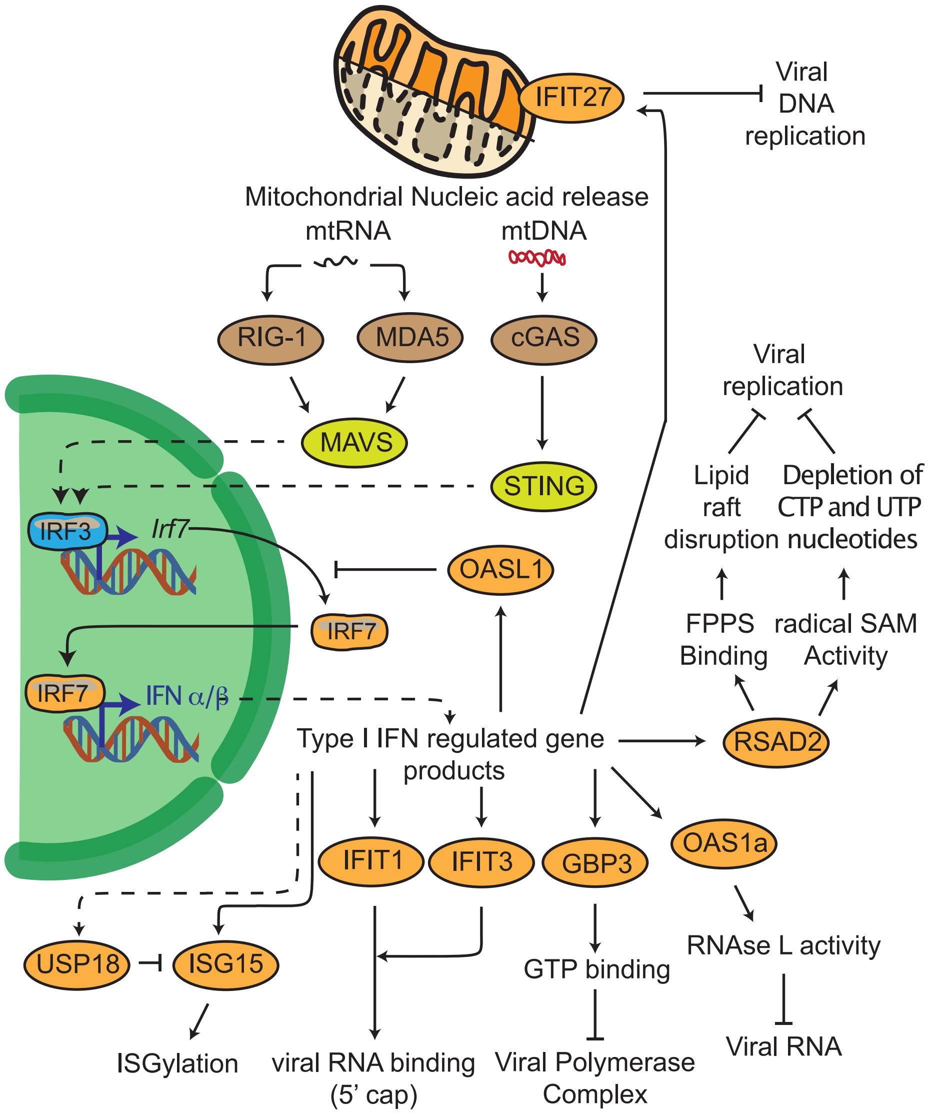

b

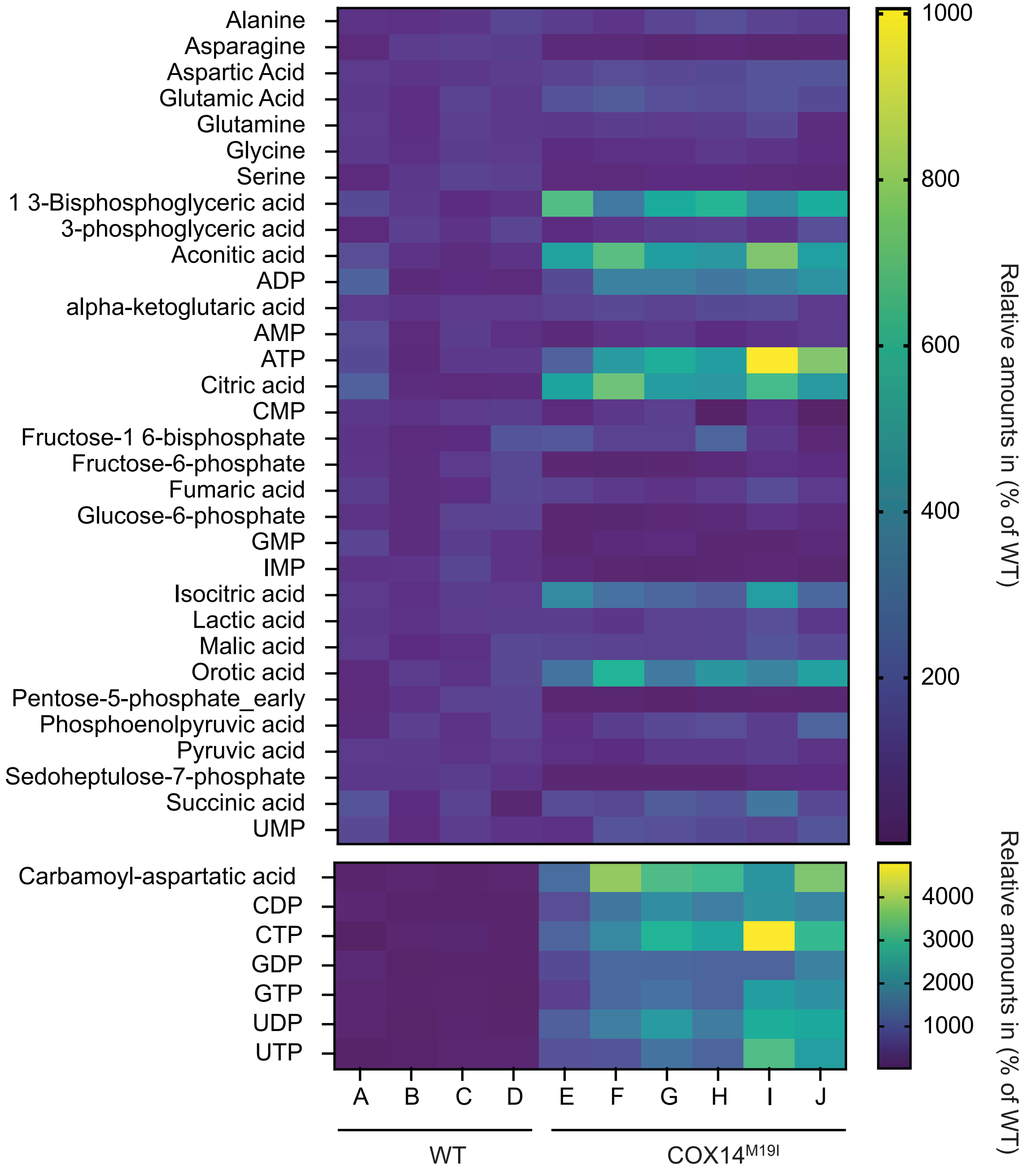

Supplementary Figure 7

Supplementary Figure 7. Signalling pathways activation in COX14^{M19I}

a Schematic presentation of pathways for mitochondrial nucleic acid sensing and subsequent activation of type I IFN pathway leading to expression of different Interferon Stimulated Genes (ISGs). **b** Heatmap depicting relative levels of different classes of metabolites determined by mass spectrometry in wild-type (WT) and COX14^{M19I} mice liver samples. n=4 (WT); n=6 (COX14^{M19I}).

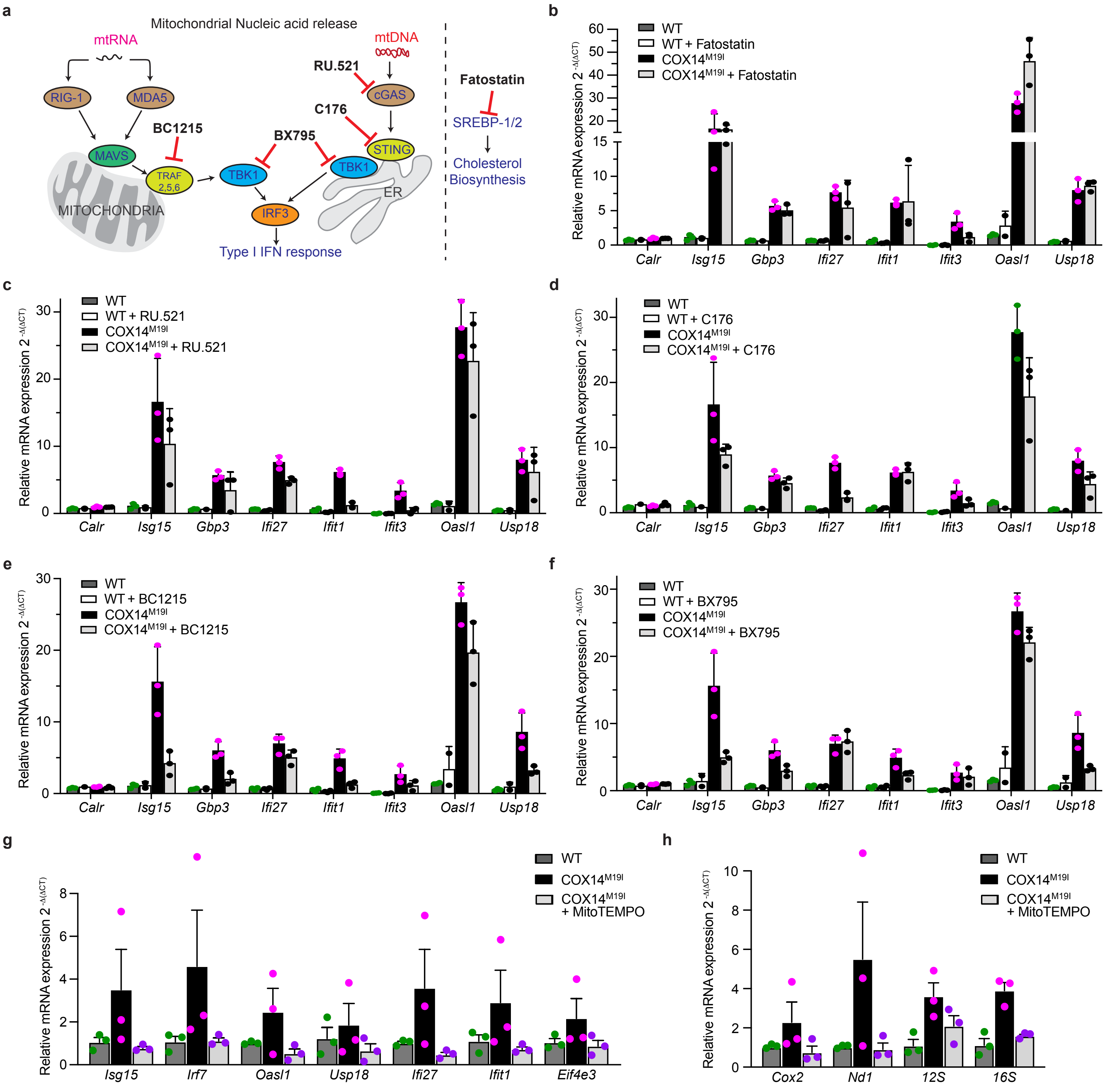

Supplementary Figure 8

Supplementary Figure 8. Pharmacological analysis of signalling processes in COX14^{M19I}

a Schematic presentation depicting targets for inhibitors of cytosolic nucleic acid sensing pathways. **b** qPCR analysis of gene expression of nucleic acid sensing pathway target genes in total mRNA isolated from WT and COX14^{M19I} primary hepatocytes treated with either vehicle control or Fatostatin for 24h. Means ± SEM, n = 3. **c** qPCR analysis of gene expression of nucleic acid sensing pathway target genes in total mRNA isolated from WT and COX14^{M19I} primary hepatocytes treated with either vehicle control or RU.521 for 24h. Means ± SEM, n = 3. **d** qPCR analysis as in (C) using primary hepatocytes treated with either vehicle control or C176 for 24h. Means ± SEM, n = 3. **e** qPCR analysis as in (C) using primary hepatocytes treated with either vehicle control or BC1215 for 24h. Means ± SEM, n = 3. **f** qPCR analysis as in (C) using primary hepatocytes treated with either vehicle control or BX795 for 24h. Means ± SEM, n = 3. **g** qPCR analysis of gene expression of nucleic acid sensing pathway target genes in total mRNA isolated from WT and COX14^{M19I} primary hepatocytes treated with either vehicle control or MitoTEMPO for 24h. Means ± SEM, n = 3. **h** Measurement of relative mRNA abundance in the cytosolic fractions from WT and COX14^{M19I} hepatocyte fractionation samples. Means ± SEM, n = 3. Source data are provided as a Source Data file.
