# Supplementary material for: Defective mitochondrial COX1 translation due to loss of COX14 function triggers ROS-induced inflammation in mouse liver
Source: Nat Commun. 2024 Aug 12;15:6914. doi: 10.1038/s41467-024-51109-y (PMC11319346; doi:10.1038/s41467-024-51109-y)
Supplement: Supplementary file 3 — Description of Additional Supplementary Files [file 41467_2024_51109_MOESM3_ESM.pdf]

Description of Additional Supplementary files

Supplementary Data 1: Serum biochemical parameters for 16-week-old COX14^{M19I} mice.

Supplementary Data 2: Lipid species in wild-type (WT) and COX14M19I mice liver samples analyzed by mass spectrometry.
