# Supplementary material for: Defective mitochondrial COX1 translation due to loss of COX14 function triggers ROS-induced inflammation in mouse liver
Source: Nat Commun. 2024 Aug 12;15:6914. doi: 10.1038/s41467-024-51109-y (PMC11319346; doi:10.1038/s41467-024-51109-y)
Supplement: Supplementary file 6 — Reporting Summary [file 41467_2024_51109_MOESM6_ESM.pdf]

nature portfolio

Corresponding author(s): Peter Rehling

Last updated by author(s): 25/07/24

Reporting Summary

Nature Portfolio wishes to improve the reproducibility of the work that we publish. This form provides structure for consistency and transparency in reporting. For further information on Nature Portfolio policies, see our Editorial Policies and the Editorial Policy Checklist.

Statistics

For all statistical analyses, confirm that the following items are present in the figure legend, table legend, main text, or Methods section.

n/a	Confirmed	
☐	☒	The exact sample size (*n*) for each experimental group/condition, given as a discrete number and unit of measurement
☐	☒	A statement on whether measurements were taken from distinct samples or whether the same sample was measured repeatedly
☐	☒	The statistical test(s) used AND whether they are one- or two-sided<br>*Only common tests should be described solely by name; describe more complex techniques in the Methods section.*
☒	☐	A description of all covariates tested
☒	☐	A description of any assumptions or corrections, such as tests of normality and adjustment for multiple comparisons
☐	☒	A full description of the statistical parameters including central tendency (e.g. means) or other basic estimates (e.g. regression coefficient) AND variation (e.g. standard deviation) or associated estimates of uncertainty (e.g. confidence intervals)
☐	☒	For null hypothesis testing, the test statistic (e.g. *F*, *t*, *r*) with confidence intervals, effect sizes, degrees of freedom and *P* value noted<br>*Give P values as exact values whenever suitable.*
☒	☐	For Bayesian analysis, information on the choice of priors and Markov chain Monte Carlo settings
☒	☐	For hierarchical and complex designs, identification of the appropriate level for tests and full reporting of outcomes
☒	☐	Estimates of effect sizes (e.g. Cohen's *d*, Pearson's *r*), indicating how they were calculated

Our web collection on statistics for biologists contains articles on many of the points above.

Software and code

Policy information about availability of computer code

Data collection	Data collection was done using softwares associated with the instruments used for specific experiments, as described in the materials and methods section
Data analysis	Western blot quantifications: ImageStudioLite Software (Version 5.2.5)<br>Numerical data: Graph Pad Prism (Version 10.0.3)<br>Proteomics: Perseus software and R Studio (version 1.4.1106)<br>RNA sequencing: Illumina's bcl2fastq (v2.20.0), FastQC (v 0.11.9), STAR (v2.7.3a), R (version 4.1.0)<br>Lipidomics: LipidView 1.3 beta (Sciex), LipidXplorer 1.2.8.1 (https://zenodo.org/record/357046923,24)

For manuscripts utilizing custom algorithms or software that are central to the research but not yet described in published literature, software must be made available to editors and reviewers. We strongly encourage code deposition in a community repository (e.g. GitHub). See the Nature Portfolio guidelines for submitting code & software for further information.

Data

Policy information about availability of data

All manuscripts must include a data availability statement. This statement should provide the following information, where applicable:

- Accession codes, unique identifiers, or web links for publicly available datasets
- A description of any restrictions on data availability
- For clinical datasets or third party data, please ensure that the statement adheres to our policy

The authors declare that all data supporting the findings of this study are available within the paper [and its supplementary files]. The corresponding author can be contacted for any additional information.
The transcriptomics data generated in this study have been deposited in the GEO database under accession code GSE269535 [https://www.ncbi.nlm.nih.gov/geo/query/acc.cgi?acc=GSE269535]
The proteomics data generated in this study have been deposited in the PRIDE database under accession code: PXD041783 [https://www.ebi.ac.uk/pride/archive/projects/PXD041783]
The Metabolomics data generated in this study have been deposited in the Zenodo database under accession code: doi (10.5281/zenodo.10640936) [https://zenodo.org/records/10640936].
The Lipidomics reporting checklists generated in this study have been deposited in the Zenodo database under accession code: doi (10.5281/zenodo.10891305) [https://zenodo.org/records/10891306], (10.5281/zenodo.10891308) [https://zenodo.org/records/10891308]. The Lipidomics MS raw data generated in this study has been deposited in the Metabolights database under accession code (MTBLS9823) [https://www.ebi.ac.uk/metabolights/MTBLS9823].
Source data are provided with this manuscript.

Research involving human participants, their data, or biological material

Policy information about studies with human participants or human data. See also policy information about sex, gender (identity/presentation), and sexual orientation and race, ethnicity and racism.

Reporting on sex and gender	*Use the terms sex (biological attribute) and gender (shaped by social and cultural circumstances) carefully in order to avoid confusing both terms. Indicate if findings apply to only one sex or gender; describe whether sex and gender were considered in study design; whether sex and/or gender was determined based on self-reporting or assigned and methods used. Provide in the source data disaggregated sex and gender data, where this information has been collected, and if consent has been obtained for sharing of individual-level data; provide overall numbers in this Reporting Summary. Please state if this information has not been collected. Report sex- and gender-based analyses where performed, justify reasons for lack of sex- and gender-based analysis.*
Reporting on race, ethnicity, or other socially relevant groupings	*Please specify the socially constructed or socially relevant categorization variable(s) used in your manuscript and explain why they were used. Please note that such variables should not be used as proxies for other socially constructed/relevant variables (for example, race or ethnicity should not be used as a proxy for socioeconomic status). Provide clear definitions of the relevant terms used, how they were provided (by the participants/respondents, the researchers, or third parties), and the method(s) used to classify people into the different categories (e.g. self-report, census or administrative data, social media data, etc.) Please provide details about how you controlled for confounding variables in your analyses.*
Population characteristics	*Describe the covariate-relevant population characteristics of the human research participants (e.g. age, genotypic information, past and current diagnosis and treatment categories). If you filled out the behavioural & social sciences study design questions and have nothing to add here, write "See above."*
Recruitment	*Describe how participants were recruited. Outline any potential self-selection bias or other biases that may be present and how these are likely to impact results.*
Ethics oversight	*Identify the organization(s) that approved the study protocol.*

Note that full information on the approval of the study protocol must also be provided in the manuscript.

Field-specific reporting

Please select the one below that is the best fit for your research. If you are not sure, read the appropriate sections before making your selection.

☒ Life sciences ☐ Behavioural & social sciences ☐ Ecological, evolutionary & environmental sciences

For a reference copy of the document with all sections, see nature.com/documents/nr-reporting-summary-flat.pdf

Life sciences study design

All studies must disclose on these points even when the disclosure is negative.

Sample size	Sample size for each experiment was determined based on prior experience and established protocol within the field for that type of experiment.

Data exclusions	No data was excluded unless statistically calculated for the specific experiment (see raw data files)
Replication	At least three biological replicates were done for each experiment. The exact number of biological replicates for each experiment is given in the Figure legends.
Randomization	Experiments were performed by two different researchers. Male and female mice were used for all experiments. Both mutant and control groups were processed at the same time and using the same reagents
Blinding	No blinding was used

Reporting for specific materials, systems and methods

We require information from authors about some types of materials, experimental systems and methods used in many studies. Here, indicate whether each material, system or method listed is relevant to your study. If you are not sure if a list item applies to your research, read the appropriate section before selecting a response.

Materials & experimental systems

n/a	Involved in the study
☐	☒ Antibodies
☒	☐ Eukaryotic cell lines
☒	☐ Palaeontology and archaeology
☐	☒ Animals and other organisms
☒	☐ Clinical data
☒	☐ Dual use research of concern
☒	☐ Plants

Methods

n/a	Involved in the study
☒	☐ ChIP-seq
☒	☐ Flow cytometry
☒	☐ MRI-based neuroimaging

Antibodies

Antibodies used

Antibodies
Rabbit polyclonal Anti-IFIT1 antibody (ab111821) Abcam Cat# ab111821, RRID:AB_10865361; dilution 1:100; https://www.abcam.com/en-de/products/primary-antibodies/ifit1-antibody-ab111821#
Mouse monoclonal Anti-Oas1a Antibody (E-2): sc-365072 Santa Cruz Cat# sc-365072, RRID:AB_10846028; dilution: 1:1000; https://www.scbt.com/p/oas1a-antibody-e-2
Mouse monoclonal Anti-ISG15 Antibody (F-9): sc-166755 Santa Cruz Cat# sc166755, RRID:AB_2126308; dilution: 1:500; https://www.scbt.com/p/isg15-antibody-f-9
Rabbit polyclonal anti-VDAC Home-made 1:1000
Rabbit polyclonal anti-ATP5 Home-made 1:2500
Rabbit polyclonal anti-RIESKE Home-made 1:2000
Anti-Rig-I (D14G6) Rabbit mAb#3743 Cell Signaling Technologies Cat# 3743s, RRID:AB_2269233; dilution: 1:1000; https://www.cellsignal.com/products/primary-antibodies/rig-i-d14g6-rabbit-mab/3743
Anti- MDA-5 (D74E4) Rabbit mAb#5321 Cell Signaling Technologies Cat# 5321s, RRID:AB_10694490; dilution 1:1000; https://www.cellsignal.com/products/primary-antibodies/mda-5-d74e4-rabbit-mab/5321
Anti- STING (D2P2F) Rabbit mAb #13647 Cell Signaling Technologies Cat# 13647S, RRID:AB_2732796; dilution 1:100; https://www.cellsignal.com/products/primary-antibodies/sting-d2p2f-rabbit-mab/13647
Mouse monoclonal ZBP1 Antibody (H-9): sc-271483 Santa Cruz Cat# sc-271483, RRID:AB_10650130; dilution 1:500; https://www.scbt.com/p/zbp1-antibody-h-9
Rabbit polyclonal Anti-Lamin B1 antibody Abcam Cat# ab16048,RRID:AB_443298 dilution 1:500; https://www.abcam.com/en-de/products/primary-antibodies/lamin-b1-antibody-nuclear-envelope-marker-ab16048#
Mouse monoclonal Anti-MTCO1 antibody [1D6E1A8] (ab14705) Abcam Cat# ab14705, RRID:AB_2084810 dilution 1:1000; https://www.abcam.com/en-de/products/primary-antibodies/mtco1-antibody-1d6e1a8-ab14705

Validation

Commercial antibodies were validated by the corresponding companies. Please refer to the links listed above. Home-made antibodies have been validated and published in various previous publications. (https://doi.org/10.15252/emmm.202317399, doi: 10.1016/j.cell.2016.09.003, https://doi.org/10.7554/eLife.32572)

Animals and other research organisms

Policy information about studies involving animals; ARRIVE guidelines recommended for reporting animal research, and Sex and Gender in Research

Laboratory animals

Mouse: COX14M19I (C57BL/6N-Cox14em83Cecad)
Mouse: COA3Y72C (C57BL/6N-Coa3emAA2(Y72C)Preh)
All ages used are stated in the figure legends

Wild animals

The study did not involve wild animals

Reporting on sex	Findings apply to both sexes. For results differing between sexes separate results are presented in the figures. Information about the numbers of separate female and male mice was not collected.
Field-collected samples	This study did not involve field-collected samples
Ethics oversight	The guidelines from the German Animal Welfare Act and approved by the Landesamt für Verbraucherschutz und Lebensmittelsicherheit, Niedersachsen, Germany (AZ: 33.9-42502-04-14/1720) were followed for the maintenance of animals and performance of animal experiments . Genome editing for this line was performed at the in vivo Research Facility of the CECAD Research Center (University of Cologne, Germany) in compliance with the European, national and institutional guidelines and approved by the State Office of North Rhine-Westphalia, Department of Nature, Environment and Consumer Protection (LANUV NRW, Germany; animal study protocol AZ 84–02.04.2014.A372)

Note that full information on the approval of the study protocol must also be provided in the manuscript.

Plants

Seed stocks	*Report on the source of all seed stocks or other plant material used. If applicable, state the seed stock centre and catalogue number. If plant specimens were collected from the field, describe the collection location, date and sampling procedures.*
Novel plant genotypes	*Describe the methods by which all novel plant genotypes were produced. This includes those generated by transgenic approaches, gene editing, chemical/radiation-based mutagenesis and hybridization. For transgenic lines, describe the transformation method, the number of independent lines analyzed and the generation upon which experiments were performed. For gene-edited lines, describe the editor used, the endogenous sequence targeted for editing, the targeting guide RNA sequence (if applicable) and how the editor was applied.*
Authentication	*Describe any authentication procedures for each seed stock used or novel genotype generated. Describe any experiments used to assess the effect of a mutation and, where applicable, how potential secondary effects (e.g. second site T-DNA insertions, mosiacism, off-target gene editing) were examined.*
